# Determining tolerant tomato genotypes to salt stress according to physiological and morphological manner

**DOI:** 10.1093/aobpla/plad037

**Published:** 2023-12-11

**Authors:** Peyman Eynizadeh, Seid Zabihallah Ravari, Mohammad Moradi, Ali Dehghani, Hamid Dehghani

**Affiliations:** Faculty of Agriculture, Plant Genetics and Breeding Department, Tarbiat Modares University, PO Box 14115-336, Tehran, Iran; Kerman Agricultural and Natural Resources Research and Education Center, Agricultural Research, Education and Extension Organization (AREEO), Kerman, Iran; Faculty of Agriculture, Plant Genetics and Breeding Department, Tarbiat Modares University, PO Box 14115-336, Tehran, Iran; College of Biological Sciences, University of California, Davis, One Shields Avenue, Davis, CA 95616, USA; Faculty of Agriculture, Plant Genetics and Breeding Department, Tarbiat Modares University, PO Box 14115-336, Tehran, Iran; Department of Plant Sciences, College of Agricultural and Environmental Sciences, University of California, Davis, One Shields Avenue, Davis, CA 95616, USA

**Keywords:** GE clustering, heatmap, modified analytical hierarchy process, multipurpose selection, salinity stress, single plant selection

## Abstract

The tomato (*Solanum lycopersicum* L.) is an annual vegetable cultivated all over the world. It faces biotic and abiotic stresses, such as salinity, in arid and semiarid regions. Investigating the relationship between physiological and economic traits, such as fruit yield, under stress conditions is necessary to identify tolerant genotypes. This study was conducted to identify tolerant tomato families according to the relationship between several important physiological, morphological and phenological traits. Twenty S3 families were cultivated in a factorial experiment (factor1: families and factor2: normal conditions and salinity stress) based on a randomized complete block design with three replications in 2019. Twenty physiological, agronomic and fruit-quality-related traits were investigated. Analysis of variance was used to prove the existing effective genetic diversity. Genetic diversity and the relationships between traits were graphically shown using heatmap clustering. Finally, genetic parameters, such as Pearson’s correlation, trait stability index and heritability were used to calculate the mathematical value of families using the Modified Analytical Hierarchy Process. Families exhibited different behaviours under normal and stress conditions. The tolerant families responded physiologically to the salt stress. Therefore, they reduced both cell membrane degradation and photosynthesis disruption by increasing proline, lycopene, carotenoid and sugar content. Therefore, fewer reductions in morphological traits were observed in these families. The most important traits based on the selection strategy were lycopene content, K^+^/Na^+^ ratio, days to flowering and biological yield. In addition, three families, H4/T/30/1, H1/T/12/5 and H1/T/47/4, were selected as the most suitable alternatives to construct the breeding population of the next generation.

## Teaser

Investigating the relationship between physiological and economic traits, such as fruit yield, under stress conditions is necessary to identify tolerant genotypes. This study was conducted to identify tolerant tomato families according to the relationship between several important physiological, morphological and phenological traits. Finally, genetic parameters, such as Pearson's correlation, trait stability index and heritability, were used to calculate the mathematical value of investigated families using Modified Analytical Hierarchy Process. Families exhibited different behaviors under normal and stress conditions. The tolerant families responded physiologically to the salt stress. Therefore, they reduced both cell membrane degradation and photosynthesis disruption by increasing proline, lycopene, carotenoid and sugar content. Fewer reductions in morphological traits were observed in these families. The most important traits based on selection strategy were lycopene content, K+/Na+ ratio, days to flowering and biological yield.

## Introduction

The tomato is an annual vegetable that belongs to the Solanaceae family. It is consumed in several ways, including fresh, cooked and canned. It has also been used in processed products, such as juice, pulp, paste and several types of sauces. (Beutner *et al.* 2001; Kimura and Sinha 2008). Salinity is a common abiotic stress in arid and semi-arid regions, which threatens global food production and impairs crop production (Colmer *et al.* 2005; Rozema and Flowers 2008). Iran is one of the countries facing salinity stress. Almost 44 million hectare, corresponding to 26.6 % of the total land soil, have been affected by varying degrees of salt in Iran (Houshmand *et al.* 2005).

One way to prevent damage from environmental stresses, such as salinity, is to use tolerant genotypes or cultivars. To overcome this problem, various morphological, physiological and molecular changes occur in plants at different levels. As suggested by Cuartero *et al.* (2006), physiological traits should be considered when evaluating salt tolerance in tomatoes. The tolerant genotypes showed the minimum morphological changes in the face of stress due to maximum physiological changes (responses). Some of the physiological responses of tomatoes to salt stress that have been frequently investigated by scientists in recent years include ionic toxicity and osmotic stress (Gharsallah *et al.* 2016), proline biosynthesis, osmotic potential of the cytoplasm, water uptake, tissue relative water content, scavenging oxidant components (Pottosin *et al.* 2014; Wang *et al.* 2017) and K^+^/Na^+^ ratio (Genc *et al.* 2010; Yongzhe *et al.* 2018; Zeng *et al.* 2018). However, Dogan *et al.* (2010) reported that physiological traits are more informative than agronomic traits for screening salt-tolerant genotypes. Therefore, the selection of genotypes according to morphological traits related to salinity tolerance, such as yield, plant height, biological yield and fruit number, could be more efficient when combined with physiological traits (Okhovatian-Ardakani *et al.* 2010; Farissi *et al.* 2014). Hence, effective genetic diversity for different traits among genotypes is necessary for effective selection (Hosseini *et al.* 2019). After examining genetic diversity, it is important to know the traits and their inheritance model in the target population to choose the selection model (direct or indirect selection). The most important parameters for finding the best selection model are the inheritance model of the target trait, the number of target traits, and the physiological or mathematical relationships among traits. For traits with a significant G × E interaction effect, comments on genotypes or environments should be made carefully. Genotype-by-Environment clustering (GE-clustering) is a new graphical method to better show the manner of genotypes according to the investigated traits in response to environmental effects (Eynizadeh *et al.* 2018).

Therefore, the indirect selection of genotypes through related traits with higher heritability than economic traits and with high correlation with them will be more efficient than direct selection (Eynizadeh and Dehghani 2020). To make an effective indirect selection, it is necessary to identify the appropriate traits based on the characteristics of each population and to identify the best genotypes based on them. Important characteristics for recognizing appropriate traits include stability under different conditions, correlation with economic traits and other items that have been previously studied by researchers. Multivariate indices are useful tools for identifying genotypes based on measured traits. The Modified Analytical Hierarchy Process (MAHP) is one of the newest methods by which superior traits and genotypes can be identified simultaneously (Eynizadeh and Dehghani 2020; Eynizadeh *et al.* 2023).

The use of statistical methods to calculate the mathematical value of genotypes based on measured traits and optional parameters and biological interpretation of the relationship between different physiological and morphological traits can help researchers determine the best choice. The objective of this study was to select tolerant tomato families for salt stress with early ripening, high fruit yield and high fruit quality (lycopene content was considered the parameter of quality) using multivariate statistical methods.

## Material and Methods

### Plant material and experiment

Twenty-one S3 families were obtained from a breeding program with four generations of self-pollinated tomato hybrids from 2015 to 2018 (Table 1). The experiment was conducted using a factorial design (21 families and 2 conditions) based on a Randomized Complete Block Design (RCBD) with three replications and five plants in each replication in 2019. The first level of the condition factor was normal irrigation (EC = 1.5 dS m^−1^) and the second level was irrigation with salt water (EC = 12 dS m^−1^), according to Maggio *et al.* (2007). Salinization was accomplished in the eight-leaf stage when plant establishment was ensured. The soil in the pots was washed with distilled water before each irrigation with saline water to prevent salt accumulation. Irrigation with saline water was performed until the EC of the leached water reached 12 dS m^−1^.

**Table 1.** Investigated hybrids in this paper.

Code	Name	Code	Name	Code	Name
1	H1/T/54/8	8	H4/T/18/4	15	H4/T/37/1
2	H3/T/5/3	9	H3/T/36/2	16	H3/T/46/7
3	H2/T/25/4	10	H2/T/16/5	17	H4/T/39/2
4	H3/T/28/2	11	H3/T/5/3	18	H1/T/23/6
5	H4/T/12/1	12	H2/T/35/7	19	H3/T/28/7
6	H2/T/17/6	13	H2/T/44/1	20	H1/T/47/4
7	H1/T/12/5	14	H1/T/18/2	21	H4/T/30/1

Since the planting medium was in pots and the controlled glasshouse conditions were considered for plant growth, there was no difference between direct sowing and indirect culture (using plantlets and transplanting) concerning the germination rate, and due to the greater uniformity of plants in direct sowing, this method was performed. Before sowing, the seeds were sterilized with sodium hypochlorite solution (10 %) for 5 min and then in ethanol (96 %) for 1 min and thoroughly washed with sterile distilled water. Seeds were sown in plastic pots (30 cm diameter and 40 cm height) filled with Hoagland’s solution (EC = 2.5 dS m^−1^1; pH = 6.0), which was replaced with irrigation. Sandy loam soil (70 % sand, 15 % silt and 15 % clay) consisting of 700 and 125 mg kg^−1^ K and P as well as 0.12 % N, 1.21 % organic carbon and 21.1 % fixed carbon was used. It should be noted that, according to the specific soil texture used in this experiment, due to the different behaviours of different soil types regarding salinity, the results may differ in clay-textured soils.

The experiment was carried out in a glasshouse with a 14-h photoperiod, natural lighting and high-pressure sodium lamps to supply an average lighting level of 10 000 Lux, 24–28 °C mean temperature and 50–55 % relative air humidity conditions.

### Measured traits

Twenty physiological, agronomical and fruit quality-related traits, including days to 50 % flowering (DTF), days to 50 % fruit formation (DFF), plant height (H), biological yield (BY), fruit number (FN), stem diameter (SD), fruit yield per plant (Yld), total soluble solids (TSS), lycopene content (Lyc), salinity (Sal), pH, sugar content (Sug), electrolyte leakage (EL), relative water content (RWC), proline content (Prol), relative chlorophyll content (SPAD), the efficiency of photosystem II (Fv/Fm), carotenoid (CAR), K^+^/Na^+^ ratio (K/Na) and Ca^2+^/Na^+^ ratio (Ca/Na) were investigated.

Morphological traits were measured at the end of the harvest in each plant. The average of samples’ data in each replication was recorded. Picking fruits and assessing their characteristics were done in the full ripening stage (red and soft, i.e. edible stage) based on Moneruzzaman *et al.* (2009). Physiological traits related to stress tolerance, including EL, RWC, Prol, Fv/Fm, Car, K/Na and Ca/Na, were measured on leaves picked up 30 days after stress application. Different samples from each plot were taken, mixed and used as a representative of each replication.

To measure EL according to Hniličková *et al.* (2019), ten 0.5-cm^2^ leaf discs were provided from the bulked sample and submerged in 10 mL of deionized water in 20-mL screw-cap vials and kept at 25 °C in the dark overnight. The electrical conductance of the solutions was measured with a conductivity meter (Model, CR-30, Colorado, Denver instrument). Then, the samples were autoclaved and total electrical conductance was quantified after vigorously shaking the vials. All measurements were recorded at 25 °C. Electrolyte leakage of samples was estimated by Equation (1):


$$\begin{array}{l} EL\left(\%\right)=\frac{C_{1}}{C_{2}}\times 100 \end{array}$$ (1)

where *C*_1_ and *C*_2_ are the electrical conductivity of the sample solutions before and after autoclaving, respectively.

To calculate RWC, fresh weight (FW), dry weight (DW) and turgid weight (TW) of the samples were determined and RWC was calculated by Equation (2) (Henson *et al.* 1981):


$$\begin{array}{l} RWC\left(\;\%\;\right)=\frac{FW-DW}{TW-DW}\times 100 \end{array}$$ (2)

Proline content per fresh weight (μmol g^−1^) was determined according to the protocol used by Claussen (2005) in tomatoes. SPAD relative chlorophyll content was determined using the SPAD-502 instrument (Minolta Co., Ltd, Osaka, Japan). The Fv/Fm was also measured by plant stress meter PSM II.

Carotenoid content was determined according to de Carvalho *et al.* (2012) by Equation (3):


$$\begin{array}{l} CAR\;(\;\mu \;g/g)=\frac{A\times V(mL)\times 10^{4}}{A_{1\;cm}^{1\%}\times P(g)} \end{array}$$ (3)

where *A*, *V*, *P* and $A_{1\;cm}^{1\;\%\;}$ are absorbance at 450 nm, total extract volume, sample weight and 2592 (a β-carotene extinction coefficient in petroleum ether). To extract free cations, the samples were shaken in 15 mL HC1 (0.5 M) for 3 days. Na^+^, K^+^ and Ca^2+^ contents were identified using atomic absorption spectrophotometer (Perkin-Elmer) in the presence of the LaCs reagent (Dubcovsky *et al.* 1996).

Tomato Lyc content was measured according to Amiri-Rigi and Abbasi (2016) with some modifications using Equation (4):


$$\begin{array}{l} Lycopene\;content\;\left(mg\cdot g^{1}\right)\;=\;\frac{A_{503}\times \;536.9\;\times \;V}{W\;\times \;17.2\;\times \;10^{4}\times \;b\;\times \;1000} \end{array}$$ (4)

where *A*_503_ is the absorbance of the nonpolar phase at 503 nm, *b* is the path length (cm), 536.9 is the molecular weight of lycopene (g mol^–1^), *V* is the volume of the nonpolar phase (mL), *W* is sample weight (kg) and 17.2 is the molar extinction coefficient for lycopene in hexane (L mol^–1^ cm^–1^).

Tomato fruit juice was provided to use by a digital refractometer to measure TSS and express results in Brix (GMK-701 AC, Korea). Also, the pH value of the tomato juice was determined using a pH meter (HANNA HI-2020, USA). The salinity of the tomato fruit juice was determined with an electrolytic conductivity meter (Model, CR-30).

### Statistical analysis

After investigating assumptions of ANOVA, a combined ANOVA with a linear model based on RCBD was done by the model:


$$\begin{array}{l} X_{ij}=\mu +\delta_{i}+\delta_{j}+\varepsilon_{ij}+\delta_{k}+\delta_{jk}+\varepsilon_{i/jk} \end{array}$$

where μ, $\delta_{i}$, $\delta_{j}$, $\varepsilon_{ij}$, $\delta_{k}$, $\delta_{jk}$ and $\varepsilon_{i/jk}$ are total average, replication effect, genotype effect, genotype × replication effect (error one), experiment effect, genotype × experiment effect and effect of the replication/genotype × experiment, respectively.

Pearson correlation between all traits with fruit yield, fruit lycopene content and DFF was calculated.

The stability of traits in different conditions was calculated using regression analysis between trait data in two experiments, where data in normal conditions were considered as independent variables and data in stress conditions as dependent variables. The absolute regression coefficient showed the stability of the trait. The lower the value of the absolute regression coefficient, the higher the trait stability. In this paper, the index calculated by this method is called stability index (SI).

**Figure 1.**
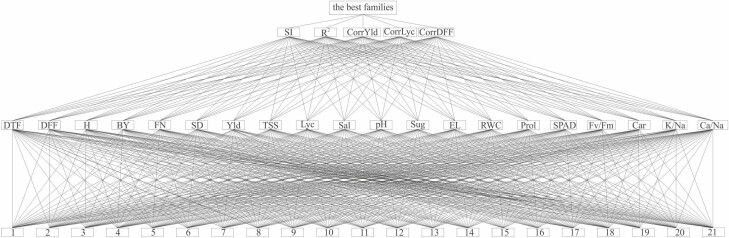
Aligning families, traits and characteristics as the first step of MAHP. DTF, days to 50 % flowering; DFF, days to 50 % fruit formation; *H*, plant height; BY, biological yield; FN, fruit number; SD, stem diameter; Yld, fruit yield; TSS, total soluble solids; Lyc, lycopene content; Sal, salinity; Sug, sugar content; EL, electrolyte leakage; RWC, relative water content; Prol, proline content; SPAD, relative chlorophyll content; Fv/Fm, efficiency of photosystem II; CAR, carotenoid; K/Na, K^+^/Na^+^ ratio; Ca/Na, Ca^2+^/Na^+^ ratio; 1–21 integers, families; SI, stability index; CorrYld, correlation with yield; CorrLyc, correlation with lycopene content; CorrDFF, correlation with days to 50 % flowering.

Cluster analysis of traits and genotype × experiment was performed, and total variation was simultaneously categorized by heatmap graphical analysis. AHP with some modifications (MAHP) according to Saaty (1987), and Eynizadeh and Dehghani (2020) was used with the following steps [**see**Supporting Information—MAHP]:

Step (1) Aligning traits and families (Figure 1).
Step (2) Calculating the weight of traits.
Step (3) Calculating the weight of families.

ANOVA, stability index, and MAHP calculations were done by MATLAB ver. 2016 (The Mathworks, Natick, MA, USA). Heatmap drawing and cluster analysis were done by Metaboanalyst 3.0. (Xia *et al.* 2009).

## Results

Table 2 shows the factorial ANOVA for the traits measured in the studied families in two conditions. As observed, the effects of genotype and experiment, and the interaction of genotype × experiment on all measured traits were significant. It means that families exhibited different responses to various traits under each condition. In other words, it was not possible to interpret the behaviour of families or the overall effect of salinity stress separately. The replication effect was not significant on any traits except stem diameter, salinity, relative chlorophyll content and K^+^/Na^+^ ratio. It revealed the uniformity of the glasshouse environment and the appropriate control of the conditions. The calculated coefficient of Determination (*R*^2^) for almost all traits was more than 0.7. The minimum value of *R*^2^ (0.33) was found in pH. As a result, the salinity effect on pH was not statistically significant.

**Table 2.** Factorial ANOVA based on RCBD. Genetic parameters of genotypic variance, phenotypic variance, broad-sense heritability, Pearson correlation with the fruit yield, Pearson correlation with the lycopene content and stability index.

S.O.V.	DF.	Mean squares									
		DTF	DFF	*H*	BY	FN	SD	Yld	TSS	Lyc	Sal
Rep.	2	47.68	76.66	42.41	76.67	3.52	4.18^*^	0.02	0.26	264.03	0.83^**^
Gen.	20	85.03^**^	183.67^**^	334.373^*^	180.31^**^	6.87^**^	5.75^**^	0.04^**^	2.48^**^	1209.45^**^	0.65^**^
En.	1	10 113.9^**^	10 690.4^**^	138 669.5^**^	55 714.9^**^	290.55^**^	600.9^**^	7.53^**^	0.05	188 535.9^**^	102.12^**^
Gen × En	20	90.43^**^	142.39^**^	644.15^**^	278.92^**^	7.62^**^	6.03^**^	0.064^**^	0.58^**^	1788.21^**^	0.78^**^
Error	82	29.04	36.82	184.98	87.19	1.29	1.02	0.01	0.24	313.32	0.15
*R* ^2^	—	0.79	0.77	0.86	0.85	0.75	0.84	0.89	0.54	0.81	0.86
CorrYld	—	−0.21	−0.16	0.64	0.56	0.75	0.53	1.00	−0.33	0.04	−0.11
CorrLyc	—	0.29	0.11	−0.02	0.16	0.08	0.28	0.04	−0.01	1.00	0.27
CorrDFF	—	0.88	1.00	0.16	0.38	−0.14	0.27	−0.16	−0.05	0.10	0.08
SI	—	0.33	0.20	0.49	0.36	0.07	0.04	0.34	0.65	0.14	0.05

S.O.V.	DF.	Mean squares									
		pH	Sug	EL	RWC	Prol	SPAD	Fv/Fm	CAR	K/Na	Ca/Na
Rep.	2	0.15	0.01	0.11	22.58	0.22	30.02^*^	9.96	0.00	25.32^*^	0.11
Gen.	20	0.24^**^	0.05^**^	1.77^**^	67.6^**^	4.94^**^	18.27^*^	45.75^**^	0.05^**^	36.97^**^	0.69^**^
En.	1	0.001	17.9^**^	0.99^**^	6618.26^**^	1166.03^**^	1900.04^**^	2438.04^**^	27.5^**^	1 3761.6^**^	0.55
Gen × En	20	0.24^**^	0.05^**^	1.08^**^	76.77^**^	2.62^**^	26.33^**^	22.43^**^	0.06^**^	31.78^**^	0.35^*^
Error	82	0.09	0.01	0.20	26.38	0.28	9.10	9.15	0.01	7.96	0.18
*R* ^2^	—	0.33	0.94	0.62	0.63	0.98	0.75	0.76	0.96	0.93	0.96
CorrYld	—	0.17	0.11	−0.56	0.72	0.10	0.55	0.69	0.66	0.53	0.49
CorrLyc	—	−0.15	0.05	−0.17	−0.11	0.29	0.00	0.00	0.25	−0.19	−0.21
CorrDFF	—	0.09	0.05	0.07	−0.15	0.21	0.28	0.01	0.01	0.25	−0.25
SI	—	0.00	0.03	0.14	0.08	0.17	0.23	0.52	0.01	1.20	0.70

^*^,

^**^significant at 5 % and 1 %, respectively; DTF, days to 50 % flowering; DF, degree of freedom; DFF, days to 50 % fruit formation; H, plant height; BY, biological yield; FN, fruit number; SD, stem diameter; Yld, fruit yield; TSS, total soluble solids; Lyc, lycopene content; Sal, salinity; Sug, sugar content; EL, electrolyte leakage; RWC, relative water content; Prol, proline content; S.O.V., source of variation; SPAD, relative chlorophyll content; Fv/Fm, efficiency of photosystem II; CAR, carotenoid; K/Na, K^+^/Na^+^ ratio; Ca/Na, Ca^2+^/Na^+^ ratio.

According to the different families’ reactions to salinity stress and the significant interaction between genotype and environment, treatment combinations were compared using Duncan’s multiple range test and the related table is available on the journal’s website [**see**Supporting Information—Duncan’s Multiple Range Test]. A wide range of data were observed for different traits under normal and salt stress conditions. Salinity stress affected the performance of families, severely. Also, the families showed very different physiological responses to salinity stress. The highest fruit yield was observed in family 4 under normal conditions and the lowest number of days to ripening was found in families 15 and 2 under stress conditions. It should be noted that the difference between these families and others was statistically significant.

In general, the families with more changes in physiological manners showed fewer changes in their performance. For example, salt stress had the highest effect on families 12 and 4 which did not show significant changes in physiological traits. Also, the lowest effect of salinity stress was on the performance of families 2 and 16, which showed significant changes in the biosynthesis of proline, sugar and carotenoid. Families 2, 8 and 9 showed the best performance under salt conditions. Also, changes in fruit yield were correlated with the fruit number under stress conditions while being correlated with the stem diameter and biological yield changes under normal conditions. The best families based on early ripening, fruit yield and fruit lycopene content under normal conditions were 12, 4 and 2.

**Figure 2.**
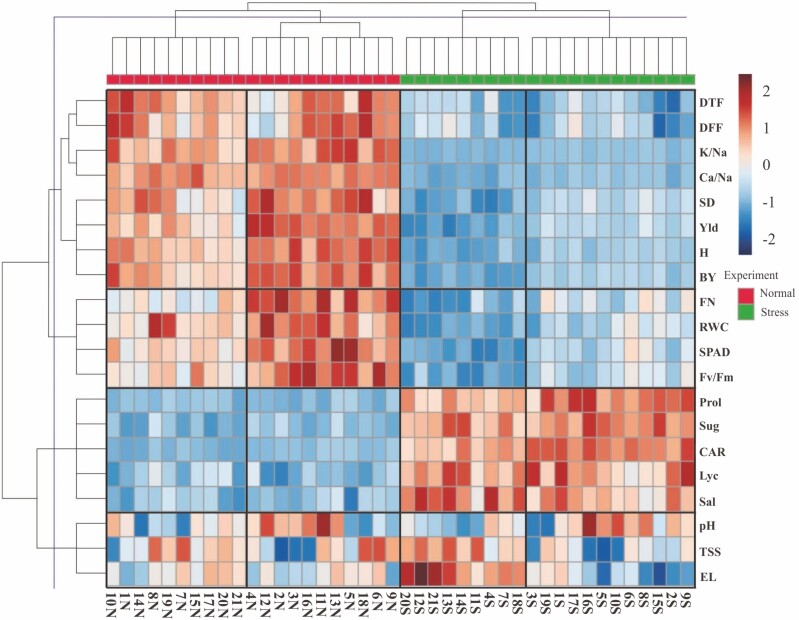
Heatmap and GE-clustering of the total variation. DTF, days to 50 % flowering; DFF, days to 50 % fruit formation; *H*, plant height; BY, biological yield; FN, fruit number; SD, stem diameter; Yld, fruit yield; TSS, total soluble solids; Lyc, lycopene content; Sal, salinity; Sug, sugar content; EL, electrolyte leakage; RWC, relative water content; Prol, proline content; SPAD, relative chlorophyll content; Fv/Fm, efficiency of photosystem II; CAR, carotenoid; K/Na, K^+^/Na^+^ ratio; Ca/Na, Ca^2+^/Na^+^ ratio; 1–21 integers, families. Bold lines indicate diversity blocks (cut lines).

According to Figure 2, the total variation was segmented into 16 blocks. Salinity stress separated families into two environments. In other words, the significant effect of salinity stress on families and their manner was exhibited by heatmap. The distribution of families in the heatmap shows the effect of artificial stress (Eynizadeh *et al.* 2021).

Traits were classified into four groups and families were classified into two groups in each experiment. The first group consisted of days to ripening, K^+^/Na^+^ ratio, Ca^2+^/Na^+^ ratio, stem diameter, fruit yield, plant height and biological yield and the second one included fruit number, relative water content, relative chlorophyll content and efficiency of photosystem II. All traits in both groups were entirely affected by stress. Measured proline, sugar, carotenoid, lycopene and salinity were classified in the third group, which increased under salinity stress. The fourth group included pH, total soluble solids and electrolyte leakage, which did not show a recognizable pattern in the two experiments. In other words, the effect of salinity stress on the last group of traits was not impressive compared to the genotype’s effect. Therefore, the only effective factor in creating diversity regarding the third group of traits is the genotype.

In normal conditions, families 3, 4, 5, 6, 9, 11, 12, 13, 16 and 18 were in a group with the highest values of morphological traits, such as yield and yield components. Another group of normal conditions consisted of families 1, 2, 7, 8, 10, 14, 15, 17, 19 and 20 with lower values of morphological traits. The two mentioned groups were similar in physiological and phenological traits under normal conditions. Families 2, 4, 5 and 12 with high amounts of yield and yield components and low amounts of phenological traits exhibited suitable agronomical characteristics under normal conditions. Considering stress conditions, families 1, 3, 5, 6, 8, 9, 10, 15, 16, 17 and 19 were in the group with the highest amounts of morphological and physiological traits. Another group included families 2, 4, 7, 11, 12, 13, 14, 17, 18 and 20 with higher values of phenological traits. The more stress-tolerant families were categorized in the first group of stress conditions. High tolerance to salinity stress in this group was due to their physiological responses, such as increased biosynthesis of proline, sugar, lycopene and carotenoid resulting in increased cell membrane resistance, increased photosynthesis and finally a better plant phenotype and fruit yield.

According to Table 3, the measured traits were weighted according to five calculated parameters. Lycopene content, K^+^/Na^+^ ratio, ripening and biological yield were the most important traits in the selection, respectively. On the other hand, electrolyte leakage, pH, total soluble solids, relative water content and sugar content of fruit juice had the lowest effect on selection, respectively.

**Table 3.** Eigen vectors of comparison matric and weights of the traits.

Traits	SI	*R* ^2^	CorrYld	CorrLyc	CorrDFF	Weight of the traits
DTF	0.179	0.219	−0.091	0.231	0.572	**1.111**
DFF	0.109	0.213	−0.069	0.088	0.650	0.991
H	0.266	0.238	0.276	−0.016	0.104	0.868
BY	0.195	0.236	0.241	0.128	0.247	**1.047**
FN	0.038	0.208	0.323	0.064	−0.091	0.542
SD	0.022	0.233	0.229	0.223	0.175	0.882
Yld	0.185	0.247	0.431	0.032	−0.104	0.790
TSS	0.353	0.150	−0.142	−0.008	−0.032	0.320
Lyc	0.076	0.225	0.017	0.797	0.065	**1.180**
Sal	0.027	0.238	−0.047	0.215	0.052	0.485
pH	0.001	0.091	0.073	−0.120	0.058	0.105
Sug	0.016	0.261	0.047	0.040	0.032	0.397
EL	0.076	0.172	−0.241	−0.135	0.045	−0.084
RWC	0.043	0.175	0.310	−0.088	−0.097	0.343
Prol	0.092	0.272	0.043	0.231	0.136	0.775
SPAD	0.125	0.208	0.237	0.001	0.182	0.753
Fv/Fm	0.282	0.211	0.298	0.001	0.006	0.798
CAR	0.005	0.266	0.285	0.199	0.006	0.762
K/Na	0.651	0.258	0.229	−0.151	0.162	**1.149**
Ca/Na	0.380	0.266	0.211	−0.167	−0.162	0.528

DTF, days to 50 % flowering; DFF, days to 50 % fruit formation; H, plant height; BY, biological yield; FN, fruit number; SD, stem diameter; Yld, fruit yield; TSS, total soluble solids; Lyc, lycopene content; Sal, salinity; Sug, sugar content; EL, electrolyte leakage; RWC, relative water content; Prol, proline content; SPAD, relative chlorophyll content; Fv/Fm, efficiency of photosystem II; CAR, carotenoid; K/Na, K^+^/Na^+^ ratio; Ca/Na, Ca^2+^/Na^+^ ratio; SI, stability index; CorrYld, correlation with yield; CorrLyc, correlation with lycopene content; CorrDFF, correlation with days to 50 % flowering. Bolded values are the greatest weights as well as the most effective traits in selection from among measured traits.

The importance of traits was assessed based on their total effects on target traits, including fruit yield, days to ripening and fruit lycopene content, determined by Pierson’s correlation. The stability index introduced in this study was another parameter that had an impact on their importance. In other words, a multivariate selection was done for multiple purposes.

Based on these weights, the families were sorted using a capability index (Table 4). Families 21, 7 and 20 were determined as the best families to create the breeding population of the next generation.

**Table 4.** Eigen vectors of comparison matric and weight of the families (capability index).

Gen	DTF	DFF	*H*	BY	FN	SD	Yld	TSS	Lyc	Sal	pH	Sug	EL	RWC	Prol	SPAD	Fv/Fm	CAR	K/Na	Ca/Na	CI
1	0.32	0.26	0.18	0.02	0.05	0.05	0.12	0.00	0.25	0.06	0.04	0.06	0.09	0.05	0.04	0.24	0.12	0.15	0.23	0.37	2.02
2	0.53	0.35	0.02	0.12	0.27	0.11	0.19	0.31	0.09	0.08	0.21	0.12	0.31	0.20	0.07	0.12	0.11	0.08	0.10	0.03	2.07
3	0.21	0.17	0.23	0.29	0.03	0.03	0.21	0.39	0.17	0.32	0.15	0.23	0.09	0.02	0.40	0.15	0.18	0.26	0.07	0.07	2.29
4	0.03	0.20	0.02	0.01	0.13	0.07	0.18	0.01	0.20	0.30	0.14	0.03	0.00	0.11	0.25	0.12	0.11	0.10	0.05	0.35	1.49
5	0.12	0.14	0.16	0.18	0.25	0.23	0.18	0.31	0.16	0.27	0.03	0.16	0.23	0.11	0.00	0.44	0.23	0.21	0.37	0.32	2.50
6	0.08	0.11	0.30	0.02	0.14	0.12	0.23	0.19	0.32	0.08	0.04	0.06	0.03	0.20	0.12	0.28	0.45	0.17	0.24	0.21	2.36
7	0.34	0.37	0.13	0.53	0.39	0.41	0.11	0.22	0.26	0.02	0.21	0.01	0.23	0.32	0.06	0.34	0.33	0.09	0.10	0.13	**3.20**
8	0.07	0.13	0.03	0.06	0.02	0.32	0.03	0.14	0.01	0.30	0.10	0.34	0.20	0.41	0.08	0.19	0.06	0.13	0.24	0.26	1.62
9	0.04	0.04	0.31	0.21	0.39	0.03	0.30	0.12	0.46	0.07	0.10	0.07	0.34	0.16	0.28	0.23	0.25	0.40	0.19	0.21	2.79
10	0.30	0.37	0.18	0.37	0.27	0.02	0.02	0.38	0.37	0.24	0.33	0.13	0.09	0.05	0.02	0.17	0.06	0.08	0.28	0.05	2.54
11	0.01	0.06	0.10	0.04	0.37	0.20	0.06	0.25	0.29	0.20	0.14	0.29	0.02	0.21	0.21	0.17	0.09	0.19	0.22	0.03	1.94
12	0.25	0.23	0.10	0.01	0.03	0.14	0.12	0.08	0.10	0.31	0.14	0.15	0.36	0.17	0.27	0.10	0.04	0.22	0.04	0.03	1.72
13	0.21	0.36	0.07	0.21	0.19	0.04	0.21	0.20	0.22	0.45	0.00	0.45	0.30	0.10	0.16	0.25	0.13	0.23	0.29	0.25	2.74
14	0.21	0.17	0.31	0.06	0.25	0.15	0.14	0.03	0.24	0.09	0.48	0.13	0.01	0.35	0.15	0.28	0.36	0.06	0.17	0.04	2.52
15	0.26	0.30	0.02	0.12	0.05	0.17	0.18	0.09	0.07	0.20	0.03	0.33	0.31	0.09	0.20	0.01	0.20	0.06	0.05	0.39	1.79
16	0.18	0.15	0.17	0.04	0.19	0.30	0.20	0.41	0.14	0.14	0.50	0.23	0.21	0.18	0.32	0.17	0.23	0.26	0.21	0.12	2.38
17	0.26	0.24	0.19	0.08	0.25	0.08	0.21	0.03	0.18	0.11	0.04	0.33	0.07	0.16	0.33	0.02	0.12	0.04	0.03	0.07	2.04
18	0.14	0.11	0.25	0.17	0.05	0.31	0.06	0.22	0.04	0.27	0.24	0.27	0.17	0.20	0.07	0.10	0.14	0.18	0.20	0.07	1.92
19	0.03	0.05	0.04	0.08	0.07	0.14	0.25	0.13	0.09	0.27	0.37	0.03	0.02	0.25	0.32	0.01	0.09	0.16	0.16	0.02	1.53
20	0.03	0.14	0.40	0.31	0.19	0.26	0.37	0.18	0.10	0.07	0.06	0.23	0.35	0.26	0.07	0.33	0.30	0.49	0.35	0.24	**3.07**
21	0.03	0.00	0.49	0.44	0.26	0.50	0.53	0.11	0.20	0.02	0.12	0.21	0.32	0.38	0.37	0.24	0.33	0.34	0.40	0.40	**3.81**

DTF, days to 50 % flowering; DFF, days to 50 % fruit formation; H, plant height; BY, biological yield; FN, fruit number; SD, stem diameter; Yld, fruit yield; TSS, total soluble solids; Lyc, lycopene content; Sal, salinity; Sug, sugar content; EL, electrolyte leakage; RWC, relative water content; Prol, proline content; SPAD, relative chlorophyll content; Fv/Fm, efficiency of photosystem II; CAR, carotenoid; K/Na, K^+^/Na^+^ ratio; Ca/Na, Ca^2+^/Na^+^ ratio; 1–21 integers, families. Bolded values are the greatest scores from among all families.

These families were determined as tolerant by heatmap, GE-clustering and MAHP as the best families for breeding to make the next generation population and gain premature salt-tolerant families with high fruit yield and high fruit quality.

## Discussion

Comparing different characteristics of a single genotype as well as comparing differences between families regarding a single trait can be exhibited using a heatmap (Eynizadeh *et al.* 2021). As seen in Figure 2, the families showed different behaviour against salt stress. Two groups, including morphological, phenological and yield-related traits, decreased and one of the groups, including physiological traits related to stress increased. Stress tolerance in plants leads to changes in plant physiological and molecular characteristics. The appearance of one or a combination of these inherent changes indicates the plant’s ability to withstand stress.

Plants almost respond to stress by regulating the osmotic pressure at the cellular level, which is characterized by an increase in osmolality. These osmolytes are organic compounds, such as proline and sugar, that do not interfere with cell metabolism. Osmotic regulation of cells leads to an increase in photosynthesis and biomass accumulation. Therefore, genotypes with a high potential for osmotic pressure regulation are more tolerant and will have higher performance under stress conditions. Osmotic regulation has been shown to provide suitable conditions for better displacement and breakdown of accumulated carbohydrates before flowering throughout the grain-filling period. In addition, maintaining high turbulence leads to greater photosynthesis and growth (Subbarao *et al.* 2000). According to Figure 2, families 16 and 8 showed the highest amounts of proline and sugar contents under salt stress.

The efficiency of Photosystem II is a normalized ratio created by dividing variable fluorescence by maximum fluorescence. This ratio represents the maximum potential quantum efficiency of Photosystem II if all reaction centres are open. An Fv/Fm value in the range of 0.79 to 0.84 is the approximate optimal value for many plant species, with lowered values indicating plant stress (Kitajima and Butler 1975; Maxwell and Johnson 2000). In the present study, this parameter was different between tolerant and sensitive families. It also showed a correlation between the relative chlorophyll content and relative water content in normal and stress conditions. Hu *et al.* (2013) showed this coordination in soybean that supplemental UV-B radiation negatively affected chlorophyll content and electron transfer activity in Photosystem II, thereby reducing the photosynthesis efficiency. It seems that families with the potential of maintaining relative water content under stress conditions had a greater ability than other families to maintain chlorophyll under stress conditions, and as a result, they had a higher photosynthesis efficiency.

Carotenes are a key part of the plant’s antioxidant defence system (Wahid 2007), but they are very sensitive to oxidative damage. The beta-carotene in the chloroplasts of all green plants binds strongly to the complex centre of photosystems I and II. Therefore, protection from the destructive effects of ROSs in this area is essential to keep chloroplasts active. Here, in addition to the role of side pigment as an effective antioxidant, beta-carotene plays a unique role in protecting photochemical processes and their stability (Havaux 1998). Biosynthesis of α-carotene and β-carotene is done in the carotenoid pathway from the conversion of lycopene by lycopene β-cyclase. The roles and underlying mechanisms of this gene in plant responses to abiotic stresses are rarely known. Kang *et al.* (2018) revealed that enhancing the biosynthesis of carotenoid from lycopene leads to abiotic stress tolerance in transgenic sweet potatoes. In this study, an increase in the amount of lycopene and carotenoids was observed under salt stress, and this increase was more in some families, such as family 1, where despite the effect of stress on early ripening, a slight drop in fruit yield was observed.

Biological membranes are the first destructive target of abiotic stresses. The integrity and stability of membranes under stress have been proven to be one of the most important factors in plant tolerance (Bajji *et al.* 2002). Membrane stability against membrane degradation is widely used as a physiological indicator to assess drought stress tolerance. It should also be noted that membrane stability is a genetic phenomenon that is inherited quantitatively (Tripathy *et al.* 2000). Electrolyte leakage is one of the traits assessed to determine membrane stability and salt stress tolerance in families. It has been stated that reducing cell volume is a factor in density and increasing the viscosity of cytoplasmic components, which increases the chances of molecules reacting, resulting in protein degradation and damaging membranes. A variety of biological compounds, including sucrose and proline, have been identified to inhibit these reactions (Hoekstra *et al.* 2001). Ashraf and Ali (2008) reported electrolyte leakage from plasma membranes as one of the most important factors to identify salt-tolerant plants. In this experiment, the electrolyte leakage of leaves progressively increased with increasing NaCl concentrations, similar to previous results (Mahmoudi *et al.* 2011; Hniličková *et al.* 2019).

Relative water content and leaf water potential are important indicators that show the relationship between water and plant. The relative water content of wheat leaves is high in the early stages of leaf development and decreases with leaf maturity and dry matter accumulation. In wheat and rice, it has been proved that the relative water content of plants exposed to stress is lower than that of plants without stress. Therefore, with the exposure of these plants to stress, leaf water potential, and relative water content decrease, and with this decrease, leaf temperature increases (Siddique *et al.* 2000). The present experiment on tomatoes and other experiments revealed a greater reduction in the relative water content of salt-sensitive cultivars as compared with tolerant ones under salt stress. Families 1, 16 and 8 had the most relative water content among other families.

Proline improves salt stress tolerance in various plant species. Under high salt conditions, proline application increases plant growth by increasing seed germination, biomass, photosynthesis, gas exchange and grain yield. These positive effects are mainly due to better nutrient uptake, water uptake and biological nitrogen fixation. It has been shown that the exogenous proline reduces salt stress intensity by increasing antioxidant activity and reducing the uptake and transport of Na^+^ and Cl^−^ while increasing the absorption of K^+^ by plants (El Moukhtari *et al.* 2020). Salinity leads to an increase in Na^+^ and Cl^−^ and it decreases Ca^2+^, K^+^, Mg^2+^, NO^3−^ and other essential nutrients (Manchanda and Garg 2008). Maintaining ion homeostasis under saline conditions is one of the adaptation strategies that tolerant plants use to produce salt stress simultaneously. These strategies may help the plant to avoid the potentially toxic effects of the accumulation of ions, such as Na^+^ and Cl^−^, which cause various types of damage to lipids, proteins and nucleic acids (Zhu and Gong 2014). Thus, Ca^2+^/Na^+^ and K^+^/Na^+^ ratios are important characteristics to distinguish tolerant families from salinity stress.

Under stress conditions, the genotypes became extremely premature. Early ripening and a decrease in the life period were quite obvious. The tomato can take advantage of the escape mechanism, which helps plants to complete their physiological growth in a short period. Thus, plants can ensure the survival of their generation by producing progenies. Flowering time is one of the most important traits associated with adaptation to drought stress. A plant with early flowering shortens the growth period and leads to escape from stress (Araus 2002). The effect of genotype and environment determined the length of the plant growth period, and these two effects determined the ability of the plant to escape environmental stresses (Dingkuhn and Asch 1999). Therefore, to achieve a high yield, the length of the plant growth period must be proportional to the soil moisture content. According to Table 2, the effect of these sources of variation along with their interaction effect was significant. Thus, the development of cultivars with a short growth period can be an effective strategy to minimize yield reduction in stress conditions (Kumar and Abbo 2001). According to Figure 2 and data means, compared to treatment combinations, the most severe effect of salt stress in early ripening was on families of 18 and 1. Also, the most stable families against stress for this parameter were 12 and 4.

The response of plants to salinity and drought is often similar. For example, the first stage of salinity stress, the osmotic effect, is quite similar to drought stress (Uddin *et al.* 2016). In other words, one of the effects of salinity stress on plants at the first stage is physiological drought. It has been found that, in drought stress, reducing plant growth, higher leaf ageing and changes in the distribution of adsorbed material among plant organs can be the main cause of reduction in fruit yield. Plant growth occurs through cell division, cell elongation and differentiation, which include genetic, physiological, ecological and morphological events and the complex interactions between these factors. The quality and quantity of plant growth depend on factors that are also affected by stress (Farooq *et al.* 2009). Therefore, in higher plants under stress, cell elongation is stopped due to a reduction in torque pressure. Also, salinity stress reduces metabolites and photosynthetic products required for cell division, which ultimately damages the mitotic process, cell elongation and growth. Thus, the decline in fruit yield observed in the studied families may be due to a significant reduction in morphological traits associated with vegetative growth such as plant height, biological yield, fruit number and stem diameter. According to Figure 2, families 18 and 5 were the most sensitive genotypes to stress based on mentioned traits. On the other hand, the lowest changes were found in families 8 and 16.

As mentioned above, the most tolerant families to salt stress were 16, 8 and 1, and the most sensitive ones were 4 and 12. Based on MAHP, the best families for selection and constructing the next generation were 21, 7 and 20 in addition to the tolerant families. Detected families by MAHP were not extreme in interesting traits of fruit yield, lycopene content and early ripening, and also they were not among the most tolerant families. However, they were the best families based on the breeding strategy (selecting tolerant genotypes to salt stress with high fruit yield, lycopene content and early ripening). They were more likely to produce suitable progenies than other families based on the breeding strategy.

## Conclusion

Salinity stress had significant effects on investigated families. ANOVA table, heatmap and GE-clustering revealed the different manners of families in face of the salt stress. Morphological characteristics and especially traits related to fruit yield were severely reduced under salt stress. The decreased yield of stress-tolerant families was less than others due to the appropriate physiological responses. Tolerant families reduced degradation of the cell membrane and disruption of photosynthesis by increasing biosynthesis of proline, lycopene, carotenoids and sugar, under salinity stress to reduce the effect of salinity on plant vigour. Stress-tolerant families were distinguished from sensitive families by different multivariate methods as families 16, 8 and 1. The most important traits for breeding strategy were identified by MAHP as lycopene content, K^+^/Na^+^ ratio, days to flowering and biological yield. Families of 21, 7 and 20 were identified as the best choices for breeding and creating the next-generation population. It was explained that selecting these families may lead to increasing the possibility of achieving premature salt-tolerant families with high fruit yield and quality.

## Supporting Information

The following additional information is available in the online version of this article –

MAHP. MATLAB script for calculating MAHP.

Duncan’s Multiple Range Test.

plad037_suppl_Supplementary_Material

plad037_suppl_Supplementary_Material_1

## Data Availability

The data and material can be made available upon request from the corresponding author Dr Hamid Dehghani at hdehghani@ucdavis.edu or dehghanr@modares.ac.ir.

## Notes

**Phenome, Genome and Environment. Chief Editor:** Colleen Doherty
