# Supplementary material for: Determining tolerant tomato genotypes to salt stress according to physiological and morphological manner
Source: AoB Plants. 2023 Dec 11;15(6):plad037. doi: 10.1093/aobpla/plad037 (PMC10712217; doi:10.1093/aobpla/plad037)
Supplement: plad037_suppl_Supplementary_Material [file plad037_suppl_supplementary_material.pdf]

Duncan's Multiple Range Test

En	Gen	En * Gen	DTF	DFF	H	BY	FN	SD	Yld	TSS	Lyc	Sal
1	1	101	99.18 pqrs	108.77 rstu	156.34 hijk	91.4 abc	8.53 ijk	12.06 efg	0.98 ef	5.27 ab	185.42 abc	3.19 b
1	2	102	81.81 defgh	90.57 jkl	145.35 fghi	101.5 defgh	13.01 tu	13.2 fghi	1.26 ghi	4.11 a	163.82 a	3.44 bc
1	3	103	89.61 hijkl	100.19 nop	171.29 jk	108.04 hij	11.29 opq	12.64 efg	1.29 hij	4.26 a	179.85 abc	3.37 b
1	4	104	81.29 defgh	87.06 hij	155.33 ghij	103.55 efghi	11.97 pqrs	13.82 ghi	1.39 ij	5.55 ab	221.92 def	3.52 bc
1	5	105	83.73 efghi	104.99 pqrs	152.93 fghi	95.05 d	12.54 rst	14.15 hij	1.22 fghi	5.01 ab	183.96 abc	2.89 a
1	6	106	92.2 jklmn	102.77 pqr	159.01 hijk	87.67 abc	10.93 nop	10.78 ef	1.27 ghij	6.43 c	194.26 abcd	3.84 bcd
1	7	107	83.74 efghi	88.8 ijk	131.63 defg	74.94 ab	7.52 efg	10.08 ef	1.07 efg	6.42 c	179.89 abc	3.72 bc
1	8	108	94.38 lmnop	91.77 jkl	144.65 efgh	92.89 abcd	7.9 fgh	13.64 fghi	0.98 ef	6.33 bc	225.53 defg	3.75 bc
1	9	109	91.68 jklm	102.03 pqr	166.39 ijk	100.69 defg	12.13 rs	11.77 efg	1.25 fghi	6.02 bc	206.61 cde	3.7 bc
1	10	110	95.54 mnopqr	110.41 stuv	154.51 fghi	107.35 fghij	8.08 ghi	12.69 efg	1.1 efg	4.41 a	171.4 ab	3.46 bc
1	11	111	93.74 klmno	105.36 qrst	165.45 hijk	98.48 def	12.88 st	12.46 efg	1.24 fghi	5.93 bc	188.77 abcd	3.95 bcd
1	12	112	78.08 cdefg	82.44 efgh	157 hijk	104.66 efghij	11.66 pqr	15.33 ij	1.38 ghij	5.03 ab	170.77 ab	3.65 bc
1	13	113	94 lmnop	107.89 qrstu	158.66 hijk	105.32 fghij	9.69 klmn	13.71 fghi	1.19 fgh	5.6 ab	191.04 abcd	4.19 cde
1	14	114	92.92 klmn	103.81 pqrs	139.61 defgh	96.38 de	9.26 klm	13.98 ghij	1.15 efg	5.31 ab	199.38 abcd	3.79 bcd
1	15	115	88.22 ghijk	97.04 mno	138.68 defg	79.43 ab	8.27 hij	10.23 ef	0.93 ef	5.37 ab	211.01 cde	3.85 bcd
1	16	116	94.93 lmnopq	106.41 qrst	136.8 defg	89.21 abc	10.62 mno	13.41 fghi	1.2 fghi	4.28 a	203.08 bcd	3.6 bc
1	17	117	91.3 jklm	101.48 opq	122.3 de	83.39 ab	7.66 fgh	11.33 ef	0.92 ef	5.78 ab	215.5 cdef	3.91 bcd
1	18	118	99.65 pqrs	111.49 stuvw	169.51 jk	109.5 hij	10.29 mn	15.19 ghij	1.13 efg	6.4 c	175.27 ab	3.88 bcd
1	19	119	90.41 ijklm	99.03 nop	131.59 defg	84.11 ab	8.86 jkl	13.05 fgh	1.18 efg	5.81 ab	200.93 bcd	4.16 cde
1	20	120	87.19 ghijk	93.35 jklm	128.25 def	81.51 ab	10.14 mn	10.78 ef	1.03 ef	5.94 bc	218.74 cdef	3.25 b
1	21	121	85.65 fghij	95.57 mn	118.33 d	77.66 ab	9.37 klmn	9.17 de	0.83 de	5.59 ab	174.49 ab	3.11 b
2	1	201	73.34 abcd	84.74 fghi	85.43 abc	53.01 ab	8.16 ghi	9.09 de	0.73 cd	5.6 ab	310.69 ijklmn	6.06 ghi
2	2	202	62.47 a	72.81 bc	84.98 abc	50.27 ab	6.78 de	8.48 bcd	0.68 bcd	5.03 ab	289.25 ghijk	5.84 fgh
2	3	203	65.45 ab	72.29 b	73.56 abc	52.28 ab	6.2 c	7.84 abc	0.66 bcd	4.41 a	306.24 hijklmn	4.75 cdef
2	4	204	79.72 cdefg	83.92 fgh	72.4 abc	42.72 ab	6.48 cd	6.26 a	0.54 abc	5.27 ab	267.45 efgh	6.42 hij
2	5	205	74.33 abcde	82.3 efgh	87.46 abc	59.42 ab	7.1 def	8.58 bcd	0.71 cd	4.11 a	259.59 defgh	5.36 efgh
2	6	206	72.33 abcd	83.2 fgh	90.99 abc	59.04 ab	7.65 fgh	8.91 cd	0.69 bcd	5.55 ab	229.34 defg	4.97 def
2	7	207	66.98 ab	73.93 bcd	88.48 abc	43.69 ab	5.97 bc	7.01 abc	0.65 bcd	5.73 ab	251.85 defg	5.27 defg
2	8	208	69.76 abc	82.68 efgh	86.36 abc	55.82 ab	9.1 klm	9.88 de	0.83 ef	5.37 ab	239.96 defg	4.43 cde
2	9	209	71.69 abc	76.65 cde	84.11 abc	55.38 ab	8.78 jk	9.2 de	0.77 de	5.59 ab	315.74 jklmno	5.14 defg
2	10	210	76.43 bcdef	88.34 ijk	87.26 abc	56.95 ab	6.56 cd	8.25 bcd	0.68 bcd	4.28 a	245.38 defg	4.88 def
2	11	211	68.52 ab	78.19 def	70.53 abc	49.06 ab	7.9 fgh	6.55 a	0.6 abc	6.4 c	239.08 defg	4.51 cdef
2	12	212	75.5 bcde	87.13 hij	65.09 a	41.22 a	5.79 b	6.65 ab	0.5 ab	6.33 c	281.3 fghij	6.33 ghi
2	13	213	74.99 abcde	90.18 jkl	75.24 abc	51.08 ab	5.66 ab	7.36 abc	0.45 a	6.42 c	301.7 hijklm	6.19 ghi
2	14	214	75.94 bcde	85.38 ghi	67.26 ab	46.26 ab	5.53 ab	8.1 bcd	0.54 abc	5.77 ab	295.42 ghijkl	5.53 fgh

Duncan's Multiple Range Test: continue

En	Gen	En * Gen	DTF	DFF	H	BY	FN	SD	Yld	TSS	Lyc	Sal
2	15	215	65.03 ab	69.08 a	91.52 bc	60.23 ab	8.49 hijk	8.99 cd	0.73 cd	5.01 ab	244.29 defg	4.62 cdef
2	16	216	73.04 abcd	81.41 efg	79.94 abc	54.16 ab	8.38 hij	9.96 ef	0.74 de	4.26 a	279.13 fghij	5.04 defg
2	17	217	79.34 cdefg	91.15 jkl	93.24 c	57.93 ab	7.2 ef	8.71 cd	0.72 cd	5.31 ab	271.96 efghi	5.47 fgh
2	18	218	66.99 ab	74.74 cd	76.79 abc	44.83 ab	7.43 efg	8.22 bcd	0.62 abc	5.78 ab	284.31 fghij	5.99 fgh
2	19	219	70.58 abc	79.22 def	94.21 c	57.57 ab	9.02 kl	8.87 cd	0.8 de	5.81 ab	251.8 defg	5.7 fgh
2	20	220	73.76 abcd	80.61 efg	72.38 abc	48.22 ab	5.26 a	7.65 abc	0.49 a	6.02 bc	257.54 defgh	5.61 fgh
2	21	221	75.2 bcde	85.0 ghi	76.27 abc	45.86 ab	5.36 a	7.21 abc	0.57 abc	5.94 bc	264.27 efgh	5.9 fgh

Duncan's Multiple Range Test: continue

En	Gen	En * Gen	pH	Sug	EL	RWC	Prol	SPAD	Fv/Fm	Car	K/Na	Ca/Na
1	1	101	4.43 cde	0.73 a	5.95 bc	69.1 bcd	2.93 cd	38.35 abc	25.09 bcd	0.67 a	22.54 ef	5.54 e
1	2	102	4.57 efgh	1.03 abc	6.12 bc	75.4 cde	2.35 ab	41.86 cd	32.11 de	0.79 bcd	26.59 fgh	7.08 jk
1	3	103	4.56 defg	0.86 ab	6.27 bc	78.6 de	2.06 b	44.88 de	34.19 def	0.78 bcd	29.24 hi	7.04 ijk
1	4	104	4.48 cdef	0.99 abc	6.07 bc	73.9 cde	2.63 bc	44.44 de	28 cd	0.73 bc	30.51 hij	6.07 ef
1	5	105	4.06 bc	0.85 ab	6.76 cdef	77.3 cde	3.51 defg	48.93 def	33.64 def	0.83 bcd	35.22 kl	7.13 jk
1	6	106	4.28 bcd	0.85 ab	7.05 efgh	71.73 cd	2.18 ab	42 cd	35.82 def	0.76 bc	32.62 ijk	6.94 hij
1	7	107	3.96 b	0.76 a	7.02 defg	68.6 bcd	2.87 bcd	39.3 bc	23.59 abc	0.81 bcd	26.14 fgh	6.87 hij
1	8	108	4.24 bc	1.1 abc	6.7 cde	82.6 def	2.54 bc	41.81 cd	27.85 cd	0.82 bcd	21.37 e	7.19 kl
1	9	109	4.12 bc	0.8 ab	5.86 bc	76.21 cde	3.3 de	43.8 cde	30.98 cde	0.83 bcd	31.34 ij	7.02 ij
1	10	110	4.59 fgh	0.91 abc	6.62 bcd	66.7 abc	2.43 b	43.26 cde	25.96 bcd	0.71 bc	33.71 jk	6.4 efg
1	11	111	4.98 ghi	0.84 ab	6.66 cde	81.2 def	2.14 ab	43.82 cde	31.06 cde	0.85 cde	33.08 jk	6.79 hi
1	12	112	4.8 ghi	0.88 ab	6.74 cdef	84.3 def	2.57 bc	45.53 def	29.07 cde	0.75 bc	30.02 hi	6.63 fgh
1	13	113	4.67 fgh	1.05 abc	7.08 fgh	72.71 cd	3.56 efg	49.29 def	33.58 de	0.85 cde	34.51 kl	6.17 efg
1	14	114	3.92 a	0.76 a	6.13 bc	66.31 abc	2.91 cd	39.77 bcd	24.23 abc	0.69 b	24.88 efg	6.65 fgh
1	15	115	4.45 cdef	0.9 abc	6.56 bcd	69.8 bcd	3.08 cde	41.42 cd	31.33 cde	0.76 bc	27.98 fgh	7.53 kl
1	16	116	4.72 fgh	0.79 a	6.23 bc	78.1 de	2.82 bc	46.17 def	35.79 def	0.74 bc	23.34 ef	6.82 hi
1	17	117	4.34 bcd	0.71 a	7.16 fgh	70.71 cd	3.19 cde	41.35 bcd	27.19 bcd	0.72 bc	27.34 fgh	6.07 ef
1	18	118	4 b	0.89 ab	6.78 cdef	67.79 bcd	2.64 bc	44.28 cde	28.8 cd	0.75 bc	24.22 efg	6.7 fgh
1	19	119	4.18 bc	0.91 abc	6.63 bcd	79.8 def	3.39 def	41.05 bcd	28.24 cd	0.71 b	30.88 hij	6.49 fgh
1	20	120	4.52 def	0.83 ab	7.11 fgh	72.2 cd	3.04 cde	38.56 abc	25.42 bcd	0.68 b	20.76 e	5.84 e
1	21	121	4.38 bcd	0.77 a	6.85 defg	67.4 bc	1.98 a	40.66 bcd	23.88 abc	0.68 b	20.21 e	5.89 e
2	1	201	4.43 cde	1.71 cde	6.99 defg	62.48 abc	9.01 kl	35.92 ab	21.69 ab	1.92 ghi	7.4 abc	2.38 ab
2	2	202	4.59 fgh	1.56 cd	5.71 b	63.84 abc	9.84 lm	34.32 ab	20.35 ab	1.59 defg	6.06 a	2.05 a
2	3	203	3.96 b	1.44 bcd	6.66 cde	55.28 a	6.35 h	35.48 ab	20.16 ab	1.9 ghi	7.26 abc	2.21 a
2	4	204	4.56 defg	1.47 bcd	7.27 fgh	55.84 a	6.98 hi	31.61 a	19.03 ab	1.63 defg	5.64 a	1.89 a
2	5	205	4.67 fgh	1.77 de	5.46 b	59.12 ab	8.14 jk	35.94 ab	21.93 abc	1.8 fgh	7.95 bc	2.87 bcd
2	6	206	4.57 fgh	1.59 cd	6.44 bcd	67.44 bcd	8.44 jkl	40.42 bcd	25.06 bc	1.84 fgh	7.56 abc	2.74 bcd
2	7	207	4.45 cdef	1.74 de	7.45 fgh	54.88 a	8.23 jkl	33.34 a	18.17 a	1.72 fgh	6.7 ab	2.57 abc
2	8	208	4.72 fgh	1.67 cde	5.67 b	62.88 abc	9.74 lm	39.12 abc	23.55 abc	1.75 fgh	8.34 bcd	2.63 abc
2	9	209	4.48 def	1.63 cde	5.83 b	61.84 abc	10.57 n	37.86 abc	25.08 bcd	1.97 hij	7.87 abc	2.66 abc
2	10	210	4.8 ghi	1.69 cde	6.32 bcd	64.96 abc	9.39 kl	37.33 abc	22.48 abc	1.67 defg	7.49 abc	2.5 abc
2	11	211	4.06 bc	1.4 bc	6.8 def	58.17 ab	7.78 j	31.45 a	16.51 a	1.43 cdef	6.79 ab	2.15 a
2	12	212	4.24 bcd	1.49 bcd	8.36 hij	53.92 a	6.84 hi	33.96 ab	21.74 ab	1.5 def	5.74 a	2.33 ab
2	13	213	4.12 bc	1.81 def	7.72 ghi	57.39 ab	9.32 kl	32.61 a	19.5 ab	1.39 cdef	6.92 ab	2.1 a
2	14	214	4 bc	1.84 def	7.26 fgh	56.36 ab	7.52 j	33.91 ab	16.71 a	1.81 fgh	6.2 a	2.27 ab

Duncan's Multiple Range Test: continue

En	Gen	En * Gen	pH	Sug	EL	RWC	Prol	SPAD	Fv/Fm	Car	K/Na	Ca/Na
2	15	215	4.28 bcd	1.86 def	5.29 a	66.08 abc	10.2 n	36.44 abc	23.5 abc	1.74 fgh	8.07 bcd	2.69 bc
2	16	216	4.98 hij	1.89 def	6.1 bc	60.32 ab	11.39 o	34.44 ab	19.77 ab	1.94 ghi	7.04 abc	2.59 abc
2	17	217	4.52 defg	1.51 bcd	6.53 bcd	57.76 ab	11.14 o	36.3 abc	19.6 ab	1.69 efgh	8.21 bcd	2.77 bcd
2	18	218	4.34 bcd	1.38 abc	7.38 fgh	59.27 ab	8.4 jkl	32.23 a	17.56 a	1.54 def	6.36 a	2.55 abc
2	19	219	3.92 b	1.6 cd	6.81 def	60.97 ab	10.84 n	36.81 abc	23.94 abc	1.88 fgh	7.64 abc	2.61 abc
2	20	220	4.38 cde	1.47 bcd	7.95 ghi	53.05 a	9.17 kl	34.28 ab	16.96 a	1.35 cde	6.48 ab	2.46 ab
2	21	221	4.18 bc	1.55 cd	8.08 ghi	54.23 a	6.61 hi	33.66 a	17.79 a	1.47 cdef	5.85 a	1.93 a
